# Supplementary material for: Dimethyl Bisphenolate Ameliorates Carbon Tetrachloride-Induced Liver Injury by Regulating Oxidative Stress-Related Genes
Source: Molecules. 2023 Dec 7;28(24):7989. doi: 10.3390/molecules28247989 (PMC10746066; doi:10.3390/molecules28247989)
Supplement: Supplementary file 1 [file molecules-28-07989-s001.zip › molecules-2637768-supplementary.pdf]

Supplemental Data

1. The targets of SEA, and SuperPhred in Supplemental Tables S1, S2, and file of SMILES.

2. The cytotoxicity assessment of DMB.

3. Supplemental methods.

1. The targets of SEA, and SuperPhred in Supplemental Tables S1, S2, and file of SMILES.

Supplemental Table S1. The targets of SEA in this study

Query ID	Target ID	Affinity Threshold (nM)	P-Value	Max Tc	Cut Sum	Z-Score	Name	Description	Query Smiles
compound_1	A0A0C7ACN7_PSEAI	5	1.20E-06	0.2807	0.2807	10.1813	pqsD	3-oxoacyl-ACP synthase	COC(=O)\C=C\C1=CC=C(O)C(O)=C1\C(=C\C1=CC(O)=C(O)C=C1)C(=O)OC
compound_1	A4_HUMAN	5	1.81E-24	0.3871	16.5761	42.1741	APP	Amyloid-beta precursor protein	COC(=O)\C=C\C1=CC=C(O)C(O)=C1\C(=C\C1=CC(O)=C(O)C=C1)C(=O)OC
compound_1	AK1BA_HUMAN	5	1.83E-23	0.3684	3.4388	40.3728	AKR1B10	Aldo-keto reductase family 1 member B10	COC(=O)\C=C\C1=CC=C(O)C(O)=C1\C(=C\C1=CC(O)=C(O)C=C1)C(=O)OC
compound_1	AK1C4_HUMAN	5	1.40E-08	0.3559	0.3559	13.6487	AKR1C4	Aldo-keto reductase family 1 member C4	COC(=O)\C=C\C1=CC=C(O)C(O)=C1\C(=C\C1=CC(O)=C(O)C=C1)C(=O)OC
compound_1	ALDR_HUMAN	5	3.06E-07	0.3684	3.7996	11.246	AKR1B1	Aldo-keto reductase family 1 member B1	COC(=O)\C=C\C1=CC=C(O)C(O)=C1\C(=C\C1=CC(O)=C(O)C=C1)C(=O)OC
compound_1	ALDR_RAT	5	2.83E-11	0.3559	5.3218	18.4868	Akr1b1	Aldo-keto reductase family 1 member B1	COC(=O)\C=C\C1=CC=C(O)C(O)=C1\C(=C\C1=CC(O)=C(O)C=C1)C(=O)OC
compound_1	AOFB_MOUSE	5	4.56E-08	0.3167	0.3167	12.7294	Maob	Amine oxidase [flavin-containing] B	COC(=O)\C=C\C1=CC=C(O)C(O)=C1\C(=C\C1=CC(O)=C(O)C=C1)C(=O)OC
compound_1	B4URF0_I33A0	5	4.62E-09	0.3333	3.0877	14.5147		Neuraminidase	COC(=O)\C=C\C1=CC=C(O)C(O)=C1\C(=C\C1=CC(O)=C(O)C=C1)C(=O)OC
compound_1	CAH13_MOUSE	5	1.96E-12	0.3438	1.5865	20.5709	Ca13	Carbonic anhydrase 13	COC(=O)\C=C\C1=CC=C(O)C(O)=C1\C(=C\C1=CC(O)=C(O)C=C1)C(=O)OC
compound_1	CAH14_HUMAN	5	9.46E-08	0.4167	3.6236	12.1605	CA14	Carbonic anhydrase 14	COC(=O)\C=C\C1=CC=C(O)C(O)=C1\C(=C\C1=CC(O)=C(O)C=C1)C(=O)OC
compound_1	CAH5A_HUMAN	5	4.89E-08	0.3673	2.2812	12.6748	CA5A	Carbonic anhydrase 5A, mitochondrial	COC(=O)\C=C\C1=CC=C(O)C(O)=C1\C(=C\C1=CC(O)=C(O)C=C1)C(=O)OC

compound_1	CAH6_HUMAN	5	1.80E-10	0.3673	2.6266	17.0452	CA6	Carbonic anhydrase 6	COC(=O)\C=C\C1=CC=C(O)C(O)=C1\C(=C\C1=CC(O)=C(O)C=C1)C(=O)OC
compound_1	CAH5B_HUMAN	5	1.60E-09	0.3673	2.2994	15.3437	CA5B	Carbonic anhydrase 5B, mitochondrial	COC(=O)\C=C\C1=CC=C(O)C(O)=C1\C(=C\C1=CC(O)=C(O)C=C1)C(=O)OC
compound_1	CAH7_HUMAN	5	2.03E-08	0.4167	4.6487	13.3606	CA7	Carbonic anhydrase 7	COC(=O)\C=C\C1=CC=C(O)C(O)=C1\C(=C\C1=CC(O)=C(O)C=C1)C(=O)OC
compound_1	DHB3_RAT	5	9.88E-09	0.3455	0.3455	13.922	Hsd17b3	Testosterone 17-beta-dehydrogenase 3	COC(=O)\C=C\C1=CC=C(O)C(O)=C1\C(=C\C1=CC(O)=C(O)C=C1)C(=O)OC
compound_1	DYN1_HUMAN	5	8.85E-22	0.3276	1.5788	37.3467	DNM1	Dynamin-1	COC(=O)\C=C\C1=CC=C(O)C(O)=C1\C(=C\C1=CC(O)=C(O)C=C1)C(=O)OC
compound_1	HDAC1_MOUSE	5	6.61E-06	0.2857	0.5662	8.8493	Hdac1	Histone deacetylase 1	COC(=O)\C=C\C1=CC=C(O)C(O)=C1\C(=C\C1=CC(O)=C(O)C=C1)C(=O)OC
compound_1	LGUL_HUMAN	5	3.95E-23	0.3509	2.2577	39.7704	GLO1	Lactoylglutathione lyase	COC(=O)\C=C\C1=CC=C(O)C(O)=C1\C(=C\C1=CC(O)=C(O)C=C1)C(=O)OC
compound_1	LOX15_RAT	5	1.17E-30	0.3621	2.1942	53.2896	Alox15	Arachidonate 15-lipoxygenase	COC(=O)\C=C\C1=CC=C(O)C(O)=C1\C(=C\C1=CC(O)=C(O)C=C1)C(=O)OC
compound_1	LOX5_RAT	5	1.42E-20	0.4231	17.1117	35.1824	Alox5	Arachidonate 5-lipoxygenase	COC(=O)\C=C\C1=CC=C(O)C(O)=C1\C(=C\C1=CC(O)=C(O)C=C1)C(=O)OC
compound_1	LX15B_RAT	5	3.57E-65	0.3621	2.1942	115.2538	Alox15b	Arachidonate 15-lipoxygenase B	COC(=O)\C=C\C1=CC=C(O)C(O)=C1\C(=C\C1=CC(O)=C(O)C=C1)C(=O)OC
compound_1	MMP1_HUMAN	5	1.60E-07	0.4286	9.3563	11.7507	MMP1	Interstitial collagenase	COC(=O)\C=C\C1=CC=C(O)C(O)=C1\C(=C\C1=CC(O)=C(O)C=C1)C(=O)OC
compound_1	MMP2_HUMAN	5	3.01E-06	0.4286	9.7746	9.4635	MMP2	72 kDa type IV collagenase	COC(=O)\C=C\C1=CC=C(O)C(O)=C1\C(=C\C1=CC(O)=C(O)C=C1)C(=O)OC
compound_1	MMP9_HUMAN	5	5.48E-08	0.4286	10.0317	12.5868	MMP9	Matrix metalloproteinase-9	COC(=O)\C=C\C1=CC=C(O)C(O)=C1\C(=C\C1=CC(O)=C(O)C=C1)C(=O)OC
compound_1	MTSI_SPISQ	5	2.25E-20	0.3455	0.6675	34.8229	sssIM	CPG DNA methylase	COC(=O)\C=C\C1=CC=C(O)C(O)=C1\C(=C\C1=CC(O)=C(O)C=C1)C(=O)OC

compound_1	MYOC_HUMAN	5	5.25E-27	0.3333	0.3333	46.7314	MYOC	Myocilin	COC(=O)\C=C\C1=CC=C(O)C(O)=C1\C(=C\C1=CC(O)=C(O)C=C1)C(=O)OC
compound_1	NEMO_HUMAN	5	3.45E-12	0.3333	0.6364	20.128	IKBKG	NF-kappa-B essential modulator	COC(=O)\C=C\C1=CC=C(O)C(O)=C1\C(=C\C1=CC(O)=C(O)C=C1)C(=O)OC
compound_1	NF2L2_HUMAN	5	4.55E-06	0.3455	0.6788	9.1413	NFE2L2	Nuclear factor erythroid 2-related factor 2	COC(=O)\C=C\C1=CC=C(O)C(O)=C1\C(=C\C1=CC(O)=C(O)C=C1)C(=O)OC
compound_1	NFKB1_HUMAN	5	1.12E-11	0.3455	0.9623	19.2099	NFKB1	Nuclear factor NF-kappa-B p105 subunit	COC(=O)\C=C\C1=CC=C(O)C(O)=C1\C(=C\C1=CC(O)=C(O)C=C1)C(=O)OC
compound_1	O49150_SOLTU	5	2.41E-07	0.3673	0.3673	11.4312		Lipoxygenase	COC(=O)\C=C\C1=CC=C(O)C(O)=C1\C(=C\C1=CC(O)=C(O)C=C1)C(=O)OC
compound_1	P89582_9HIV2	5	1.57E-14	0.3182	0.3182	24.3352	pol	Integrase	COC(=O)\C=C\C1=CC=C(O)C(O)=C1\C(=C\C1=CC(O)=C(O)C=C1)C(=O)OC
compound_1	PAR15_HUMAN	5	3.55E-11	0.3276	0.3276	18.3103	PARP15	Protein mono-ADP-ribosyltransferase PARP15	COC(=O)\C=C\C1=CC=C(O)C(O)=C1\C(=C\C1=CC(O)=C(O)C=C1)C(=O)OC
compound_1	POL_RSVP	5	4.88E-18	0.2857	0.2857	30.6299	gag-pol	Gag-Pol polyprotein	COC(=O)\C=C\C1=CC=C(O)C(O)=C1\C(=C\C1=CC(O)=C(O)C=C1)C(=O)OC
compound_1	PPBN_HUMAN	5	5.88E-06	0.3188	0.9153	8.9407	ALPG	Alkaline phosphatase, germ cell type	COC(=O)\C=C\C1=CC=C(O)C(O)=C1\C(=C\C1=CC(O)=C(O)C=C1)C(=O)OC
compound_1	Q26964_TRYCR	5	2.83E-24	0.2985	0.2985	41.8277	TCTS-154	Trans-sialidase	COC(=O)\C=C\C1=CC=C(O)C(O)=C1\C(=C\C1=CC(O)=C(O)C=C1)C(=O)OC
compound_1	Q7ZJM1_9HIV1	5	1.88E-29	0.4231	14.8418	51.1235	pol	Integrase	COC(=O)\C=C\C1=CC=C(O)C(O)=C1\C(=C\C1=CC(O)=C(O)C=C1)C(=O)OC
compound_1	Q8HY88_BOVIN	5	5.03E-14	0.3455	0.3455	23.4246	KCNK2	Potassium channel subfamily K member 2	COC(=O)\C=C\C1=CC=C(O)C(O)=C1\C(=C\C1=CC(O)=C(O)C=C1)C(=O)OC

compound_1	Q8I2J3_PLAF7	5	7.63E-09	0.3281	1.5784	14.123		M18 aspartyl aminopeptidase	COC(=O)\C=C\C1=CC=C(O)C(O)=C1\C(=C\C1=CC(O)=C(O)C=C1)C(=O)OC
compound_1	SDF1_HUMAN	5	1.05E-63	0.3214	3.4974	112.6199	CXCL12	Stromal cell-derived factor 1	COC(=O)\C=C\C1=CC=C(O)C(O)=C1\C(=C\C1=CC(O)=C(O)C=C1)C(=O)OC
compound_1	T23O_MOUSE	5	9.88E-07	0.3333	0.3333	10.3317	Tdo2	Tryptophan 2,3-dioxygenase	COC(=O)\C=C\C1=CC=C(O)C(O)=C1\C(=C\C1=CC(O)=C(O)C=C1)C(=O)OC
compound_1	TAU_HUMAN	5	1.68E-27	0.3607	5.4569	47.6208	MAPT	Microtubule-associated protein tau	COC(=O)\C=C\C1=CC=C(O)C(O)=C1\C(=C\C1=CC(O)=C(O)C=C1)C(=O)OC
compound_1	TBB1_HUMAN	5	5.80E-09	0.3115	0.8825	14.3374	TUBB1	Tubulin beta-1 chain	COC(=O)\C=C\C1=CC=C(O)C(O)=C1\C(=C\C1=CC(O)=C(O)C=C1)C(=O)OC
compound_1	TF65_HUMAN	5	5.15E-06	0.3529	0.9372	9.0443	RELA	Transcription factor p65	COC(=O)\C=C\C1=CC=C(O)C(O)=C1\C(=C\C1=CC(O)=C(O)C=C1)C(=O)OC
compound_1	TLR4_MOUSE	5	6.56E-44	0.3455	4.2828	77.0775	Tlr4	Toll-like receptor 4	COC(=O)\C=C\C1=CC=C(O)C(O)=C1\C(=C\C1=CC(O)=C(O)C=C1)C(=O)OC
compound_1	TTHY_HUMAN	5	2.52E-07	0.3559	1.0079	11.3953	TTR	Transthyretin	COC(=O)\C=C\C1=CC=C(O)C(O)=C1\C(=C\C1=CC(O)=C(O)C=C1)C(=O)OC
compound_1	XDH_BOVIN	5	8.85E-06	0.3774	2.9565	8.6218	XDH	Xanthine dehydrogenase/oxidase	COC(=O)\C=C\C1=CC=C(O)C(O)=C1\C(=C\C1=CC(O)=C(O)C=C1)C(=O)OC
compound_1	XDH_HUMAN	5	4.16E-07	0.3016	1.4607	11.0052	XDH	Xanthine dehydrogenase/oxidase	COC(=O)\C=C\C1=CC=C(O)C(O)=C1\C(=C\C1=CC(O)=C(O)C=C1)C(=O)OC

Supplemental Table S2. The targets of SuperPhred in this study

Targets	ChEMBL-ID	UniProt ID	PDB	TTD ID	Possibility	Accuracy
DNA-(apurinic or apyrimidinic site) lyase	CHEMBL5619	P27695	6BOW	T13348	99.63%	91.11%
Monoamine oxidase A MAO	CHEMBL1951	P21397	2Z5Y	Not Available	97.73%	91.49%
Arachidonate 12-lipoxygenase ALOX12	CHEMBL3687	P18054	3D3L	Not Available	94.55%	75.57%
Tyrosyl-DNA phosphodiesterase 1 TDP1	CHEMBL1075138	Q9NUW8	6N0D	Not Available	92.28%	71.22%
Kruppel-like factor 5 KLF5	CHEMBL1293249	Q13887	Not Available	Not Available	92.16%	86.33%
Nuclear factor erythroid 2-related factor 2 NRF2	CHEMBL1075094	Q16236	2FLU	Not Available	90.94%	96%
Transthyretin TTR	CHEMBL3194	P02766	6SUG	T86462	90.61%	90.71%
Pregnane X receptor PXR	CHEMBL3401	O75469	6TFI	T82702	90.34%	94.73%
Transcription intermediary factor 1-alpha TIF-1a	CHEMBL3108638	O15164	4YBM	Not Available	89.31%	95.56%
Proteasome component C5	CHEMBL4208	P20618	6KWY	Not Available	88.97%	90%
DNA topoisomerase II alpha TOP2A	CHEMBL1806	P11388	6ZY5	T17048	88.74%	89%
Dual specificity protein kinase CLK4	CHEMBL4203	Q9HAZ1	6FYV	Not Available	88.22%	94.45%
Nuclear factor NF-kappa-B p105 subunit NK-KB P105	CHEMBL3251	P19838	1SVC	Not Available	88.13%	96.09%
Beta-glucuronidase	CHEMBL2728	P08236	3HN3	T96413	87.91%	77.68%
Signal transducer and activator of transcription 1-alpha/beta STAT1	CHEMBL6101	P42224	1YVL	T64205	84.81%	72.62%
Macrophage migration inhibitory factor MIF	CHEMBL2085	P14174	6B1K	T39977	84.74%	80.78%
Thyroid hormone receptor alpha	CHEMBL1860	P10827	3ILZ	T79591	81.74%	99.15%
Cathepsin D	CHEMBL2581	P07339	4OD9	T67102	81.32%	98.95%
Glycine transporter 2 GLYT-2	CHEMBL3060	Q9Y345	Not Available	Not Available	81.29%	99.17%
Cytochrome P450 3A4	CHEMBL340	P08684	5VCC	T37848	79.26%	91.19%
Nuclear receptor ROR-beta	CHEMBL3091268	Q92753	Not Available	Not Available	79.17%	95.50%
Cyclin-dependent kinase 5	CHEMBL4036	Q00535	4AU8	T20973	78.49%	79.09%

Endoplasmic reticulum-associated amyloid beta-peptide-binding protein	CHEMBL4159	Q99714	2O23	Not Available	77.71%	70.16%
Serine/threonine-protein kinase/endoribonuclease IRE1	CHEMBL1163101	O75460	6W39	Not Available	76.85%	98.11%
G protein-coupled receptor kinase 5	CHEMBL5678	P34947	4TND	Not Available	76.61%	88%
Protein tyrosine kinase 2 beta	CHEMBL5469	Q14289	4EKU	T07087	75.85%	91.03%
Serine/threonine-protein kinase PLK4	CHEMBL3788	O00444	3COK	Not Available	75.55%	83.65%
Glycine receptor subunit alpha-1	CHEMBL5845	P23415	4X5T	T50269	74.76%	90.71%
Platelet-derived growth factor receptor alpha	CHEMBL2007	P16234	7LBF	T53524	73.57%	91.07%
Protein-tyrosine phosphatase 2C	CHEMBL3864	Q06124	5EHR	T13057	73.40%	94.42%
G-protein coupled receptor 6	CHEMBL3714130	P46095	Not Available	Not Available	73.22%	97.36%
Cyclin-dependent kinase 2/cyclin E1	CHEMBL1907605	P24864	1W98	T70176	73.14%	92.88%
Neuronal acetylcholine receptor; alpha4/beta4	CHEMBL1907591	P30926	6UR8	T70967	72.48%	100%
PI3-kinase p110-alpha/p85-alpha	CHEMBL2111367	P27986	4JPS	T80276	72.25%	94.33%
Glucose transporter	CHEMBL2535	P11166	6THA	Not Available	71.69%	98.75%
C-C chemokine receptor type 2	CHEMBL4015	P41597	5T1A	T89988	71.60%	98.57%
Glutaminase kidney isoform, mitochondrial	CHEMBL2146302	O94925	3UO9	T86734	70.98%	100%
Excitatory amino acid transporter 1	CHEMBL3085	P43003	5LM4	Not Available	70.22%	94.67%
ADAM10	CHEMBL5028	O14672	6BE6	T31902	70%	97.50%
Sodium channel protein type III alpha subunit	CHEMBL5163	Q9NY46	Not Available	T76937	69.80%	96.90%
Proteasome Macropain subunit	CHEMBL3492	P49721	5LE5	Not Available	68.83%	90.24%
TRAF2- and NCK-interacting kinase	CHEMBL4527	Q9UKE5	2X7F	Not Available	66.99%	70%
Phosphodiesterase 3B	CHEMBL290	Q13370	1SO2	Not Available	66.59%	94%
Vascular endothelial growth factor receptor 1	CHEMBL1868	P17948	5T89	Not Available	66.56%	96.47%
Ephrin type-B receptor 2	CHEMBL3290	P29323	3ZFM	T73756	66.18%	78%
BMP-2-inducible protein kinase	CHEMBL4522	Q9NSY1	4W9W	Not Available	65.57%	78.11%
Photoreceptor-specific nuclear receptor	CHEMBL4374	Q9Y5X4	4LOG	Not Available	64.89%	85%
Casein kinase II alpha/beta	CHEMBL3038477	P67870	6TLS	T51565	64.77%	99.23%
Serotonin 2c (5-HT2c) receptor	CHEMBL225	P28335	6BQH	T83813	64.20%	89.62%
Serine/threonine protein kinase NLK	CHEMBL5364	Q9UBE8	Not Available	Not Available	63.76%	79%

Glutamate receptor ionotropic, AMPA 2	CHEMBL4016	P42262	2WJW	T42392	63.44%	86.92%
Dual specificty protein kinase CLK1	CHEMBL4224	P49759	6KHD	Not Available	62.92%	85.30%
Platelet-derived growth factor receptor	CHEMBL2095189	P09619	3MJG	T53524	62.37%	71.67%
NT-3 growth factor receptor	CHEMBL5608	Q16288	6KZD	Not Available	62.29%	95.89%
Lipoxin A4 receptor	CHEMBL4227	P25090	6OMM	Not Available	61.21%	100%
P2X purinoceptor 4	CHEMBL2104	Q99571	Not Available	T60330	61.13%	97.50%
Excitatory amino acid transporter 3	CHEMBL2721	P43005	6X2L	Not Available	61.05%	93.50%
Peptidyl-prolyl cis-trans isomerase NIMA-interacting 1	CHEMBL2288	Q13526	1PIN	T16308	60.84%	91.71%
Lysosomal Pro-X carboxypeptidase	CHEMBL2335	P42785	3N2Z	Not Available	60.61%	100%
C-C chemokine receptor type 1	CHEMBL2413	P32246	Not Available	T16016	60.45%	89.50%
Vasopressin V1b receptor	CHEMBL1921	P47901	Not Available	Not Available	60.32%	92.50%
NADPH oxidase 1	CHEMBL1287628	Q9Y5S8	Not Available	Not Available	60.06%	95.48%
Dipeptidyl peptidase II	CHEMBL3976	Q9UHL4	4EBB	Not Available	60.05%	92.29%
Serine/threonine-protein kinase TAO1	CHEMBL5261	Q7L7X3	Not Available	Not Available	59.63%	89.33%
Dual-specificity tyrosine-phosphorylation regulated kinase 1A	CHEMBL2292	Q13627	6S14	T92803	59.52%	93.24%
AMP-activated protein kinase, alpha-1 subunit	CHEMBL4045	Q13131	6C9H	Not Available	58.85%	73.50%
Muscarinic acetylcholine receptor M3	CHEMBL245	P20309	Not Available	T67684	58.77%	97.53%
Nuclear receptor subfamily 4 group A member 1	CHEMBL1293229	P22736	4RZF	Not Available	58.73%	78%
Tyrosine-protein kinase receptor RET	CHEMBL2041	P07949	6Q2O	T60631	58.56%	91.79%
Cystic fibrosis transmembrane conductance regulator	CHEMBL4051	P13569	6MSM	T55654	58.52%	95.71%
Galectin-3	CHEMBL4531	P17931	6FOF	T72038	58.46%	96.90%
Dual specificity phosphatase Cdc25B	CHEMBL4804	P30305	1QB0	Not Available	58.20%	79.50%
Cytochrome P450 2A6	CHEMBL5282	P11509	2FDV	T06455	58.03%	71.78%
Ectonucleotide pyrophosphatase/phosphodiesterase family member 1	CHEMBL5925	P22413	6WFJ	Not Available	57.47%	92.38%
G-protein coupled receptor 55	CHEMBL1075322	Q9Y2T6	Not Available	T87670	57.37%	78.15%
Excitatory amino acid transporter 2	CHEMBL4973	P43004	Not Available	Not Available	56.87%	98.75%

Glutathione S-transferase Pi	CHEMBL3902	P09211	5J41	T21669	56.64%	93.81%
Cyclin-dependent kinase 1/cyclin B1	CHEMBL1907602	P06493	6GU2	T49898	56.60%	91.24%
G-protein coupled bile acid receptor 1	CHEMBL5409	Q8TDU6	7CFM	T86273	56.34%	93.65%
Kallikrein 7	CHEMBL2443	P49862	2QXI	Not Available	55.84%	94%
Acetyl-CoA carboxylase 2	CHEMBL4829	O00763	3TDC	T08922	55.82%	98%
G-protein coupled receptor 35	CHEMBL1293267	Q9HC97	Not Available	Not Available	55.80%	89.34%
Coagulation factor XIII	CHEMBL4530	P00488	4KTY	Not Available	55.74%	96%
Arachidonate 5-lipoxygenase	CHEMBL215	P09917	3V98	Not Available	55.60%	92.68%
Formyl peptide receptor 1	CHEMBL3359	P21462	Not Available	T87831	55.58%	93.56%
Dopamine D1 receptor	CHEMBL2056	P21728	7JVP	Not Available	55.12%	91%
Ribosomal protein S6 kinase alpha 3	CHEMBL2345	P51812	4D9T	Not Available	55.06%	95.64%
Serine/threonine-protein kinase mTOR	CHEMBL2842	P42345	6BCX	T75243	55.02%	92.78%
Aldose reductase	CHEMBL1900	P15121	1US0	T26623	55%	92.38%
Acetylcholine receptor; alpha1/beta1/delta/gamma	CHEMBL1907588	P02708	5HBT	T04689	54.61%	98.33%
Serine/threonine-protein kinase ULK3	CHEMBL5047	Q6PHR2	6FDY	Not Available	54.47%	78.50%
C5a anaphylatoxin chemotactic receptor	CHEMBL2373	P21730	6C1R	T15439	54.37%	92.62%
Dual specificity protein phosphatase 3	CHEMBL2635	P51452	3F81	Not Available	54.03%	94%
Endoplasmic reticulum aminopeptidase 1	CHEMBL5939	Q9NZ08	6Q4R	Not Available	53.92%	100%
Quinone reductase 2	CHEMBL3959	P16083	4FGL	T75498	53.63%	89.49%
Activin receptor type-1B	CHEMBL5310	P36896	Not Available	Not Available	52.87%	70%
Angiotensin-converting enzyme	CHEMBL1808	P12821	5AMB	T82577	52.23%	93.39%
Tissue factor pathway inhibitor	CHEMBL3713062	P10646	5NMV	T78890	51.43%	97.33%
P2X purinoceptor 7	CHEMBL4805	Q99572	Not Available	T63414	50.98%	97.50%

SMILES data

The data of SMILEs

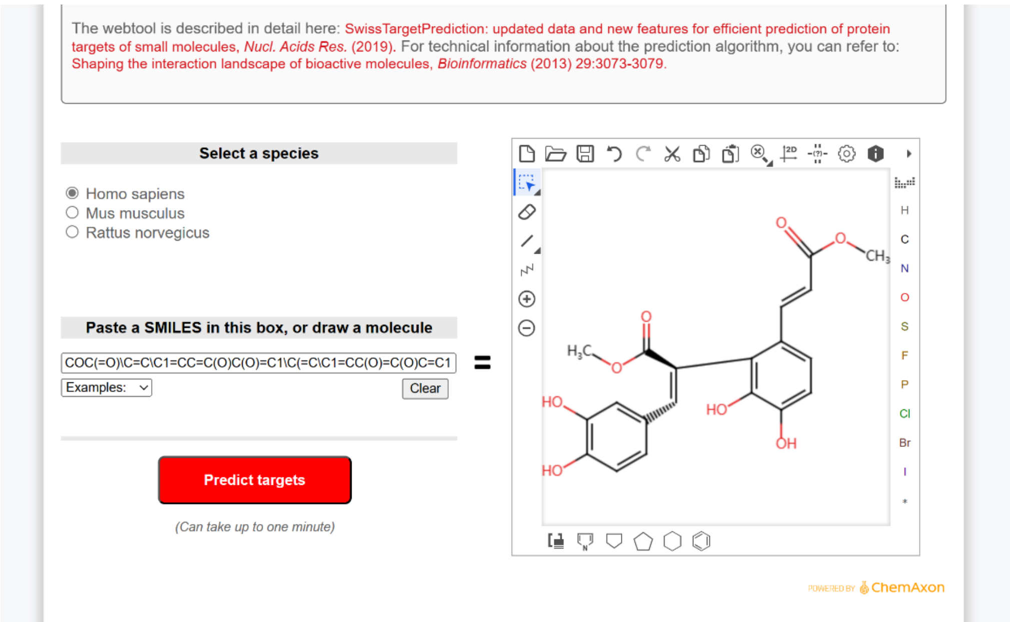

2. The cytotoxicity assessment of DMB.

We did experiment to assess the cytotoxicity of DMB in cells, and the results showed that 10, 20, 30, 60, and 100 μg/mL of DMB did not have cytotoxicity to MHCC-97H and 293T cells (Supplemental figure 1). In vitro study, treatment of 45 mg/kg DMB did not induce significant changes of liver tissues (Supplemental figure 2) through histological analysis of the liver tissues.

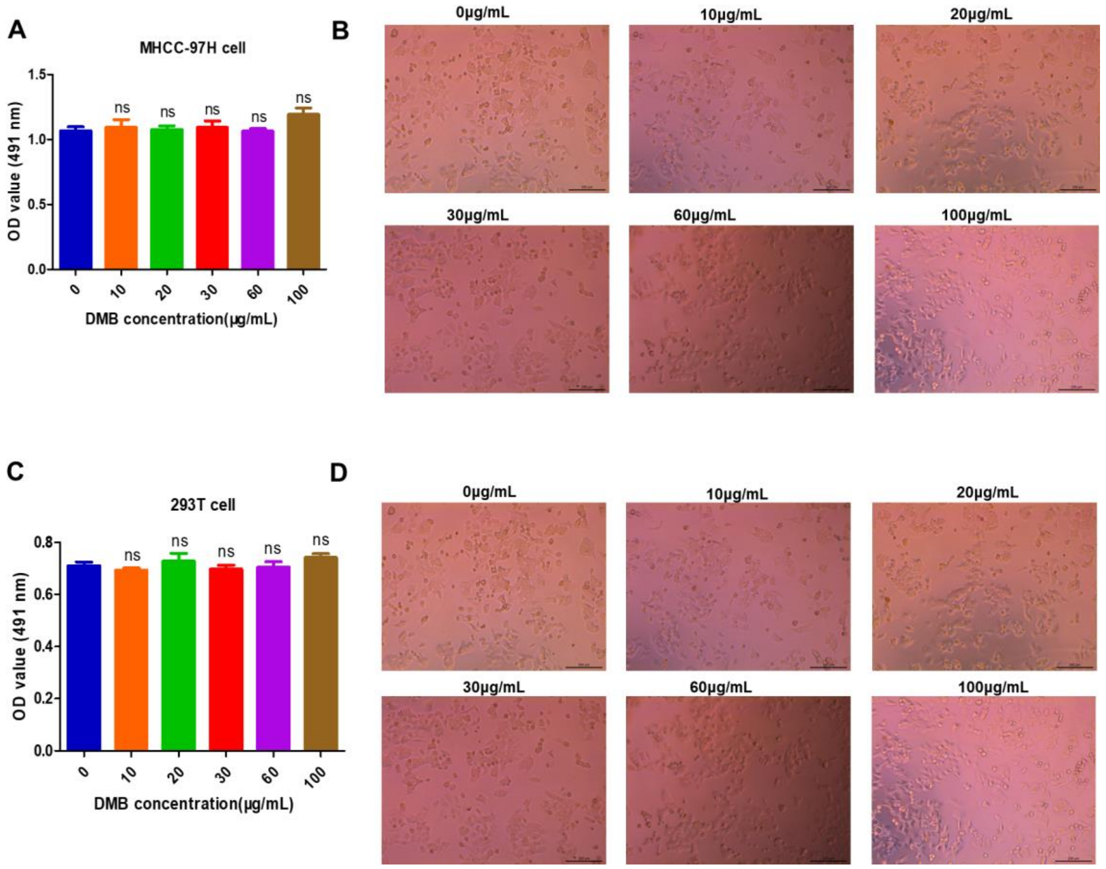

Supplemental Figure S1. Cytotoxicity analysis of DMB in vitro.

(A, B) MTT and Microscopic observation results showed that 10 to 100 μg/mL DMB did not have cytotoxicity to MHCC-97H, respectively.

(C,D) MTT and Microscopic observation results showed that 10 to 100 μg/mL of DMB did not significantly affect the growth of 293T cells, respectively.

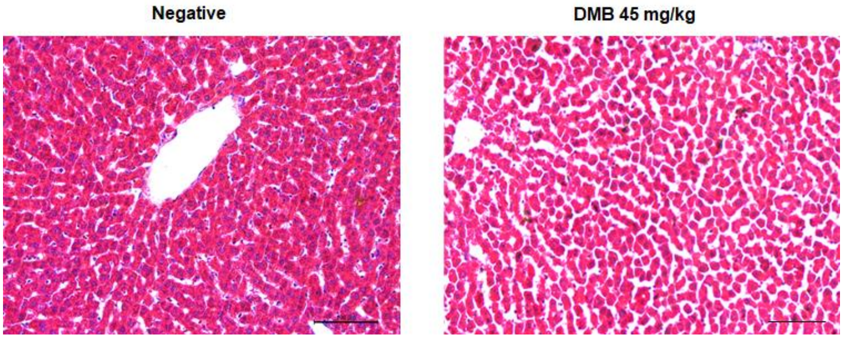

Supplemental Figure S2. Histological changes of liver tissues of model rats induced by DMB.

3. Supplemental methods

3.1 ALT analysis by alanine substrate method.

ALT detection was using the alanine substrate method with Alanine Aminotransferase Kit (H001, Meikang Biotechnology Co., Ltd.) in accordance with the manufacturer's instructions. The amino group from alanine is transferred to α-ketoglutaric acid under the catalysis of ALT, which produces pyruvic acid. Pyruvic acid reacts with NADH to produce lactic acid. NADH absorbance is detected by rate method at 340nm to reflect the ALT activity.

Mixed 200 μL reagent 1 (Trihydroxymethylaminomethane buffer, NADH, lactate dehydrogenase, sodium azide) and 15 μL sera and incubated at 37 °C, 3 min. Added another 100 μL reagent 2 (L-alanine, α-ketoglutaric acid, sodium azide), mixed well and incubated at 37 °C, 1min. The absorbance was detected at 340nm to calculate ALT activity.

3.2 AST analysis by aspartate substrate method.

AST analysis was using the aspartate substrate method with Aspartate Aminotransferase Kit (H002, Meikang Biotechnology Co., Ltd.) in accordance with the manufacturer's instructions.

AST catalyzes aspartic acid and α-ketoglutarate to produce oxaloacetic acid and glutamic acid. The oxaloacetic acid is changed to malic acid by malic dehydrogenase. At the same time, the coenzyme I (NADH) is oxidized to NAD+. the activity of AST in the sample can be calculated rate method at 340nm with a decrease in absorbance.

Mixed 200 μL reagent 1 (Trihydroxymethylaminomethane buffer (pH 7.6), α-ketoglutaric acid, NADH, LDH, MDH, sodium azide) and 15 μL sera and incubated at 37 °C, 3 min. Added another 100 μ L Reagent 2 (L-alanine, sodium azide), mixed well and incubated at 37 °C, 1.5 min. The absorbance was detected at 340nm to calculate AST activity.

3.3 Alkaline phosphatase assay

ALP analysis was using NPP substrate method with Alkaline Phosphatase Kit (H003, Meikang Biotechnology Co., Ltd.) in accordance with the manufacturer's instructions. Under the action of ALP, disodium p-nitrophenylphosphate and 2-amino-2-methyl-

propanol would generate 4-nitrophenol and 2A2M1P phosphate. With the action of ALP, disodium p-nitrophenylphosphate and H_2O would generate 4-nitrophenol and phosphate. ALP activity was calculated by assessing the generation rate of 4-nitrophenol at 405nm.

Added 6 μL sera into 240 μL Reagents 1 (2A2M1P, magnesium acetate, EDTA, zinc sulfate), mixed and incubated at 37 °C for 3 min. Added 60 mL Reagent 2 (4-NPP), mixed and incubated at 37 °C, 1 min. The absorbance was monitored at 405nm to calculate ALP activity.

3.4 Detection principle: Lactic acid substrate method

ALP analysis was using NPP substrate method with Lactate dehydrogenase Kit (H008, Meikang Biotechnology Co., Ltd.) in accordance with the manufacturer's instructions. Lactate dehydrogenase catalyzes lactic acid to produce pyruvate, and NAD^+ is reduced to NADH. The rate of increase is proportional to the LDH activity in absorbance at 340nm.

Added 6 μL sera into 200 μL Reagents 1 (N-acetyl-D-glucamine buffer, L-lithium lactate), mixed and incubated at 37 °C for 3 min. Added 100μL Reagent 2 (NAD^+), mixed and incubated at 37 °C, 1.5 min. The absorbance was monitored at 340nm to calculate LDH activity.

3.5 TBIL detection

TBIL was detected using TBIL Kit (60152901, DiaSys Diagnostic Systems GmbH, Germany) in accordance with the manufacturer's instructions. The total bilirubin in the sample with 2,4-dichloroaniline diazonium salt can form a diazo compound and turn red, which is proportional to the total bilirubin content and be detected at 546nm.

Added 25 μL sera into 1mL of reagent 1 (Phosphate buffer, sodium chloride, surfactant/stabilizer), mixed, and incubated at 37 °C, 5 min. Added another 250 μL Reagent 2 (2,4-dichloroaniline diazonium salt, hydrochloric acid, surfactant) at 37 °C, 5 min. the absorbance was read at 546nm to reflect TBIL.

3.6 DBIL detection

DBIL was detected using DBIL Kit (60152900, DiaSys Diagnostic Systems GmbH, Germany) in accordance with the manufacturer's instructions.

Added 100 μL sera into 1 mL of Reagent 1 (EDTA-Na2, sodium chloride, amino sulfonic acid), mixed, and incubated at 37 °C, 5 min. Added another 250 μL Reagent 2 (2,4-dichloroaniline diazonium salt, hydrochloric acid, EDTA-Na2) at 37 °C, 5 min. the absorbance was read at 546nm to reflect TBIL.
